# Confirmatory Factor Analysis of Knowledge, Attitude, and Behaviour Questionnaire towards Oral Health among Indian Adults

**DOI:** 10.3390/jpm11040320

**Published:** 2021-04-20

**Authors:** Siddharthan Selvaraj, Nyi Nyi Naing, Nadiah Wan-Arfah, Somasundaram Prasadh

**Affiliations:** 1 Faculty of Medicine, Medical Campus, Universiti Sultan Zainal Abidin, Kuala Terengganu, Terengganu 20400, Malaysia; sidzcristiano@gmail.com; 2 Faculty of Health Sciences, Universiti Sultan Zainal Abidin, Kuala Terengganu, Terengganu 20400, Malaysia; wanwaj@unisza.edu.my; 3 Faculty of Dentistry, National University of Singapore, Singapore 119077, Singapore; e0204949@u.nus.edu

**Keywords:** item response theory, confirmatory factor analysis, validity, knowledge, attitude and behaviour, oral health

## Abstract

**Background:** Oral health-related conditions are among the common conditions seen in adults in India. The usage of inappropriate measurement tools that are unvalidated may result in deceptive and imprecise findings that might lead to substandard plans for cessation programs and ineffectiveness. This study was conducted to validate a questionnaire that can assess the factor structure of knowledge, attitude, and behaviour towards oral health among adults in India by confirmatory factor analysis. **Methods:** Simple random sampling was conducted among adults in India. A total of 260 adults participated in this study. The knowledge, attitude, and behaviour (KAB) questionnaire on oral health was circulated among the adults who were willing to participate in the study after it was explained to them, and the questionnaires were retrieved once they completed. Software R version 3.6 was used to analyse the data of this study. Robust maximum likelihood was utilized for the assessment due to the violation of multivariate normality assumption. For attitude and behaviour domain, a three-factor model was used for measurement model validity and construct validity. **Results:** The confirmatory factor analysis of the three-factor model for the 26-item KAB questionnaire on oral health gave sufficient goodness-of-fit values and the measurement model exhibited ideal convergent and discriminant validity following model re-specification. The three-factor model was tested to obtain measurement model validity and construct validity for attitude and behaviour domains. The results of this study gave a statistically significant value (*p* < 0.001), with χ^2^ (df) values of 39 (7) and 28 (11) for attitude and behaviour domains, respectively. **Conclusions:** The KAB oral health questionnaire used in this study has a valid measurement model and reliable constructs. It was found to be an ideal tool to measure the KAB towards oral health among adults in India.

## 1. Background of the Study

Oral health-related conditions are seen in a broad range around the globe [1]. People in India face a serious disparity when comes to oral health care [2]. Conditions that are related to oral health can be avoided with ideal oral hygiene measures at home [3]. Maintaining good oral hygiene has a superior effect on ensuring better general health [4]. For the past 10 years, dental conditions have been seen more prominently among the Indian population [5]. Literacy towards oral health can have an effect on oral health outcome [6]. Oral health promotion through health education is an ideal choice that can help deliver better input on oral health for the Indian population [7].

Carrying out a study to assess the knowledge, attitude, and behaviour towards oral health is the best way to strengthen the oral health literacy of adults [8]. Oral conditions like dental caries, or cavities, are seen in broad range of individuals around the globe, but they can be prevented by utilizing an ideal strategy targeting certain populations by implementing the right tool [9]. Similarly, using a knowledge, attitude, and behaviour (KAB) questionnaire towards oral health would help achieve a positive outcome. Oral health diseases strongly influence the quality of life among young individuals [10]. Hence, it is better to strengthen the oral health status of an individual to achieve an ideal quality of life by assessing their perceptions towards oral health. An individual’s behaviour, psychological view, and attitude towards oral health are frequently ignored, though they have a vital role in identifying clinical results [11]. To identify an individual’s view of oral health, the best way is to assess their knowledge, attitude, and behaviour.

To our knowledge, until now, there are few KAB questionnaires available to assess the knowledge, attitude, and behaviour of adults towards oral health. This questionnaire was developed by reviewing various research articles to have an ideal order of questions that can lower the possibility of misunderstanding each item in questionnaire and to attain the study objective that frames the study productive. Hence, this study was conducted to determine the construct validity and reliability of the knowledge, attitude, and behaviour towards oral health among adults in India.

## 2. Methods

### 2.1. Research Design and Study Population

A cross-sectional study was conducted among Indian adults residing in Tamil Nadu. Exploratory factor analysis of the questionnaire was carried out among different population who reside in the city of Chennai, Tamil Nadu, to study the construct validity and reliability of the questionnaire. Items in the questionnaire that showed an acceptable psychometric property with good construct validity and reliability results in the first stage of this study were considered and included in the final version of the questionnaire to carry out confirmatory factor analysis.

### 2.2. Sample Size and Sampling Method

A total of 260 adults from a residential community in Chennai-Tamil Nadu participated in this study. The study participants were selected by simple random sampling. The individuals willing to participate in this study were approached and given a brief explanation of the study and the outcome of the validation process. Consent was obtained from the participants who were willing to participate. After obtaining consent from the study participants, the questionnaires were circulated and retrieved once completed by the participants. The sample size of this study was calculated based on the recommendations of a study carried out by Hair in the year 2010 for confirmatory factor analysis [12].

## 3. Measurement Tool

A self-administered questionnaire was used to assess the knowledge, attitude, and behaviour of Indian adults towards oral health. Exploratory factor analysis was carried out for all domains and sub-domains in the earlier part of this study. Table 1 shows the content and the response choice of the questionnaire. Table 2 summarizes the content of KAB questionnaire towards oral health. The questionnaire has four domains. The final version of the questionnaire consists of 39 questions that include four domains: (1) demography profile domain contains 13 questions to assess the demographic profile of participants; (2) knowledge profile domain contains 11 questions to assess the level of knowledge towards oral health; (3) attitude profile domain contains 8 questions to assess attitudes towards oral health; and (4) behaviour profile domain contains 7 questions to assess behaviours towards oral health. The sociodemographic characteristics that were surveyed include age, gender, race, religion, diet, smoking habits, alcohol habits, marital status, occupational status, level of education, income, house ownership, and vehicle ownership. The knowledge part of the questionnaire was produced based on the aetiology, risk factors, symptoms, and complications of oral-related diseases. The attitude part of the questionnaire was created based on the Health Belief Model (HBM) theory [13]. The behaviour questions were based on the preventive strategies for oral health-related conditions, endorsed by the World Health Organization and the Centres for Disease Control and Prevention.

## 4. Data Collection Procedures

Data collection was carried out between January and February 2021. The self-administered questionnaire was prepared on paper and distributed to the adults who reside in Chennai who met the inclusion criteria of this study. Individuals who were above 18 years of age, who can write and read English, and those who were volunteering to be part of this study were included. The procedures, study purpose, and confidentiality of the answers given to the questions in the study were elaborated to the participants by the primary investigator before distributing the questionnaire. Informed consent was acquired from the study participants before distributing the questionnaire. Instructions were given to the participants to provide valid and honest answers while completing the questionnaire. Once the participants finished the questionnaire, it was retrieved immediately. It took approximately 10 to 15 min to complete the questionnaire.

## 5. Data Management and Preliminary Analysis

Data were entered and missing data were checked by SPSS software version 24 and then moved to R version 3.6.0 for item response theory (IRT) and confirmatory factor analysis (CFA) analysis. Data analysis was carried out using R version 3.6.0.

## 6. Item Response Theory (IRT)

Considering on the one-dimensionality of the questions containing dichotomous responses of the knowledge, this section was analysed by two-parameter logistic item response theory (2-PL IRT) analysis, using the ltm package version 1.0.0 6.

## 7. Confirmatory Factor Analysis (CFA)

Confirmatory factor analysis (CFA) was carried out to verify the factorial structure of the KAB questionnaire identified in the EFA carried in another part of this study. The attitude and behaviour domains were analysed by lavaan package version 0.5–22 [14]. Number of indices showed a good model fit for the construct, which comprises of: the ratio of chi-square to degree of freedom (χ^2^/df) < 5.0, root mean square error of approximation (RMSEA) ≤ 0.08, comparative fit index (CFI) > 0.9, Tucker–Lewis Index (TLI) > 0.9, and *p* > 0.05 for the chi-square test [15]. To assess composite reliability, semTools package version 0.4-14 5-6 was utilized to establish Raykov’s rho [16]. Hair and his colleagues in the year 2009 put forward that model fitness can be determined by at least a minimum of three individual indices. Ideal association among items and respective factors are displayed by a regulated factor loading higher than 0.5 as well as a p-value of less than 0.05; consequently, it demonstrates the validity of the construct. Composite reliability of the domains was determined with a value of 0.7, and values above were taken as desirable [17].

## 8. Result

### Socio-Demographic Characteristics

The socio-demographic characteristics of participants are summarized in Table 3. There were 187 (70.77%) male and 76 (29.23%) female participants involved in the study. The distribution of participants among age groups of 18–24 years, 25–34 years, 35–44 years, and ≥45 years were found to be 69 (26.54%), 94 (36.15%), 64 (24.62%), and 33 (12.69%), respectively. Out of 260 participants, 182 (70%) were married. In terms of religion, 135 (51.92%), 88 (33.85%), 29 (11.15%), and 8 (3.08%) participants identified as Hindu, Muslim, Christian, and other, respectively. A total of 244 (93.85%) participants had Tamil ethnicity whereas 16 (6.15%) had an ethnicity other than Tamil. The majority of participants (56%) had a mixed type of diet. The majority of the participants were not smokers (78%) or consumers of alcohol (72%). In terms of education, 3 (1.15%), 42 (16.15%), 98 (37.69%), and 117 (45.00%) participants were illiterate, had attended primary school, had attended high school, and had attended university, respectively. In this study, 150 (57.69%), 16 (6.15%), 40 (15.38%), and 54 (20.77%) participants were employed, unemployed, students, and homemakers, respectively. Incomes of below 10 K ₹ rupees, 10 K ₹ rupees–20 K ₹ rupees, 20K ₹ rupees–30K ₹ rupees, and above 30 K ₹ rupees were reported by 109 (41.92%), 14 (5.38%), 67 (25.77%), and 70 (26.92%) participants, respectively. A total of 147 (56.54%) and 113 (43.46%) participants owned and rented houses, respectively, whereas 127 (48.85%) and 133 (51.15%) participants owned and did not own vehicles, respectively.

## 9. IRT for Knowledge-Based Questions

The sample size needed for 2-PL IRT is not specified; however, a few studies put forward a range of samples from 100 to 500 [18]. A sample size of 260 was considered for the item response theory (IRT) analysis of the knowledge domain of the questionnaire. In the knowledge section, IRT analysis results showed an acceptable range for both difficulty (−4 to +4) and the discrimination parameter on each of the items. All the items were retained as they had acceptable difficulty and discrimination values. Based on the study by Raykov and Marcoulides in the year 2016, the amount of information tapped by the items between −4 and +4 difficulty in which the difficulty range was 96.64%. The knowledge domain consists of 11 items that include: K1 (‘there are two sets of teeth during the lifetime’), K2 (‘tooth infection causes gum bleeding’), K5 (‘replacement of a missing tooth improves oral hygiene’), K6 (‘the dental caries of deciduous teeth need not be treated’), K7 (‘bacteria is one of the causes of gingival problems’), K8 (‘fizzy soft drinks affect the teeth adversely’), K9 (‘loss of teeth can interfere with speech’), K10 (‘irregularly placed teeth can be moved into correct position by dental treatment’), K11 ( ‘decayed teeth can affect the appearance of a person’), K13 (‘tobacco chewing or smoking can cause oral cancer’), K14 (‘white patches on teeth are called dental plaque’). In terms of internal consistency reliability, Cronbach’s alpha was 0.75. IRT analysis for the psychometric characteristics of the domain is shown in Table 4.

## 10. CFA of Attitude Questions

For the attitude domain, the three-factor model was then tested by CFA using an MLR estimation method in which the highest likelihood parameter was evaluated with standard errors and a chi-square test. MLR estimation was initiated in CFA models as the model implies an excess of one exploratory variable. The three-factor model showed a goodness of fit with χ^2^ [df = 17] = 39, *p* = 0.002; CFI_robust_ = 0.991; TLI_robust_ = 0.986; RMSEA_robust_ = 0.07 (0.041–0.10); SRMR = 0.016; AIC = 4593.4. The composite reliability of the factors has a satisfactory cut-off value of >0.7, as summarized in Table 5.

## 11. CFA of Behaviour Questions

For the behaviour domain, the three-factor model was tested by CFA using an MLR estimation method in which the highest likelihood parameter was evaluated with standard errors and a chi-square test. MLR estimation was initiated in CFA models as the model implies an excess of one exploratory variable. The three-factor model showed a goodness of fit with χ^2^ [df = 11] = 28, *p* = 0.003; CFI_robust_ = 0.990; TLI_robust_ = 0.981; RMSEA_robust_ = 0.07 (0.041–0.10); SRMR = 0.031; AIC = 4086. The composite reliability of the factors has a satisfactory cut-off value of >0.7 except item B7, as summarized in Table 6.

Table 7 describes the fit indices for the confirmatory factor models of knowledge and attitude domain.

## 12. Discussion

Confirmatory factor analysis is the next level of construct validity and better than exploratory factor analysis and simple reliability analysis, similar to internal consistency reliability and test-retest in various ways. CFA is similar to structural equation modelling, which is associated with model measurement [19]. CFA helps connect items to their respective domains which permit, fix measurement model associations, and put forward measures to assess the fit of the theoretical model that is suggested for data collection [20]. Hence, CFA is considered an ideal way to validate behavioural and social sciences [19].

Measurement scale development includes various protocols and procedures to institute validity and reliability. The content and quality of the primary constructs and the option of items to be incorporated can also be achieved through a pilot study or acquired from a similar study which was done earlier and validated by CFA [21]. The usage of inappropriate measurement tools which are unvalidated results in deceptive and imprecise findings might result in substandard plans for cessation programs and ineffectiveness [22]. The item response theory model makes uses of the concept of a true score model, which constitutes a group of dogmatic formulae to conduct systematic analysis that helps to attain the objective that is required [23].

In this study, a new dataset was investigated to check the appropriateness for the three-factor model devised in a previous validation study. The phases of the construction of questionnaires to evaluate the knowledge, attitudes, and behaviours of Tamil Nadu people towards oral health were recorded in the present study. Thus, using IRT and CFA, the validation of the questionnaires was achieved. In general, CFA results of knowledge, attitude, and behaviour domains showed that each construct’s measurement models are fit. The findings in our study support the initially proposed three-factor sub-domain of attitude and behaviour sections.

A good difficulty psychometric property of the knowledge domain was exhibited by IRT analysis. The ideal parameter range for discrimination values ranges from minus infinity to plus infinity; nonetheless, questions with negative figures of discrimination are recognized as problematical because they infer that participants with a high score are less expected to support more stringent response alternatives [24]. In the current study, all the selected items showed the discrimination parameters to be positive and less challenging. All the items previously screened by EFA study earlier were validated to be meaningful and made more sense to the knowledge questionnaire. Confirmatory factor analysis with maximum likelihood was used for both attitude and behaviour questionnaires. The recommendation of Cole in the year 1987 for the goodness of fit was followed; i.e., chi-square goodness-of-fit; the goodness-of-fit (GFI), and the root mean-square residual (RMSEA) [25]. In this current study, we also considered CFI, TLI, SRMR, and AIC to assess the goodness of fit of the model [26]. The three-factor model was found to have good fit to the data for both attitude and behaviour. The goodness-of-fit index was acceptably high (>0.90). The root means square residual also suggested that the model provided an acceptable fit to the data (RMSEA = 0.07). From previous studies, it was learnt that RMSEA values less than 0.05 are good, values between 0.05–0.08 are acceptable, values between 0.08–0.1 are marginal, and values greater than 0.1 are poor [27]. Other parameters like CFI and TLI should be over 0.9 for a good fit [28], and in the current study, for both the data it was found to be >0.90.

In general, factor loadings and construct reliability should be equal to or greater than 0.70 for good convergent validity [29]. From the CFA result of this study, all loadings were greater than 0.70 except one item in the behaviour data for which loading was between 0.6 and 0.70. Low convergent validity means the items have information of other factors rather than the corresponding factor alone, which means the factors are associated with one another and this can be explained as latent factors that compose one concept in the real world are always dependent [30].

Our study findings displayed an ideally good fit for the questionnaire, providing confirmatory characteristics for the factor structure for attitude and behaviour domains. Fit indices like RMSEA, CFI, TLI, and SRMR are with satisfactory values and hence have ideal construct validity [31]. The reliability of the domains was derived from Raykov’s rho and the internal consistency reliability was determined using Cronbach’s alpha. The attitude and behaviour factors of the KAB questionnaire possess ideal reliability, with coefficients exceeding 0.70. Similar to other research, this study has some limitations [32]. Data were collected from Indian adults using a simple random sampling method; consequently, the study results cannot be taken as representative of the Indian population.

## 13. Conclusions

The KAB questionnaire exhibited ideal validity, reliability, and psychometric properties while measuring the knowledge, attitude, and behaviour of adults towards oral health. The outcome of this article can be considered as a guide to conduct future studies to assess individual knowledge, attitude, and behaviours towards oral health. In addition, the developed questionnaire can be utilized to plan oral health promotion programs in the future based on the KAB towards oral health obtained using this tool and to frame intervention strategies based on the outcome.

## Figures and Tables

**Table 1:** Response Choice of KAB Questionnaire towards Oral Health.

Domains	Total Items	Measurement	Response Choice
Demography	13	Socio demographic characteristics: age, gender, education, income, ethnicity, occupation, etc.	Open-ended, closed-ended, multiple-choice
Knowledge	11	Aetiology, clinical manifestation, treatment, symptoms, preventive measures on oral health	Yes/No/I don’t know. 1 = correct answer, 0 = wrong/I don’t know
Attitude	8	Individuals’ attitudes towards oral health based on health belief model	1 = Strongly agree 2 = Agree 3 = Neither agree nor disagree 4 = Disagree 5 = Strongly disagree
Behaviour	7	Various actions towards oral hygiene that might have a good or ill effect on oral health	1 = Never 2 = Seldom 3 = Occasionally 4 = Very often 5 = Always

**Table 2:** KAB questionnaire on oral health.

Knowledge	Attitude	Behaviour
1. There are two sets of teeth during lifetime	1. Brushing teeth twice a day improves oral hygiene	1. I give importance to my teeth as much as any part of my body
2. Tooth infection causes gum bleeding	2. Keeping your teeth clean and healthy is beneficial to your health	2. I have sensitive teeth
3. Replacement of missing tooth improves oral hygiene	3. Improper brushing leads to gum disease	3. I brush my tooth twice daily
4. The dental caries of deciduous teeth need not be treated	4. Sweets retention leads to tooth decay	4. I use teeth to open cap of bottled drink
5. Bacteria is one of the reasons to cause gingival problems	5. Brushing with fluoridated toothpaste prevent tooth decay	5. I experience tooth ache while chewing food
6. Fizzy soft drinks affect the teeth adversely	6. Dentists care only about treatment & not prevention	6. I have bleeding gums during brushing
7. Loss of teeth can interfere with speech	7. Gum bleeding denotes gum infection	7. I do routine dental check-up
8. Irregularly placed teeth can be moved into correct position by dental treatment	8. Scaling is harmful for gums	
9. Decayed teeth can affect the appearance of a person		
10. Tobacco chewing, or smoking can cause oral cancer		
11. White patches on teeth are called dental plaque		

**Table 3:** Socio-demographic characteristics of study population (*n* = 260).

Parameter		*n*	%
Age	18–24 years	69	26.54
	25–34 years	94	36.15
	35–44 years	64	24.62
	≥45 years	33	12.69
Gender	Male	184	70.77
	Female	76	29.23
Marital Status	Yes	182	70.00
	No	78	30.00
Religion	Hindu	135	51.92
	Muslim	88	33.85
	Christian	29	11.15
	Others	8	3.08
Ethnicity	Tamil	244	93.85
	Others	16	6.15
Diet	Vegetarian	111	42.69
	Non-vegetarian	3	1.15
	Mixed	146	56.15
Smoking	Yes	58	22.31
	No	202	77.69
Alcohol	Yes	72	27.69
	No	187	71.92
Education	Illiterate	3	1.15
	Primary	42	16.15
	High school	98	37.69
	University	117	45.00
Employment	Employed	150	57.69
	Unemployed	16	6.15
	Student	40	15.38
	Homemaker	54	20.77
Income	Below 10 K ₹	109	41.92
	10 K ₹–20 K ₹	14	5.38
	20 K ₹–30 K ₹	67	25.77
	Above 30 K ₹	70	26.92
House	Owned	147	56.54
	Rented	113	43.46
Vehicle	Yes	127	48.85
	No	133	51.15

**Table 4:** Result of EFA for knowledge-based questionnaires.

Items	Difficulty	Discrimination	χ^2^ (df = 10)	*p* Value
K2: Tooth infection causes gum bleeding	−1.97	1.03	7.17	0.519
K7: Loss of teeth can interfere with speech	−1.97	1.08	15.08	0.058
K6: Fizzy soft drinks affect the teeth adversely	−1.89	1.18	7.33	0.501
K9: Decayed teeth can affect appearance	−1.77	1.21	8.63	0.374
K8: Irregularly placed teeth can be moved into correct position	−1.76	1.16	7.86	0.447
K11: White patches on teeth are called dental plaque	−1.62	1.48	7.86	0.447
K5: Bacteria is one of the causes of gingival problems	−1.60	1.57	6.97	0.540
K3: Replacement of a missing tooth improves oral hygiene	−1.40	2.10	16.64	0.034
K4: Dental caries of deciduous teeth need not be treated	−1.34	2.09	11.49	0.176
K10: Tobacco chewing or smoking causes oral cancer	−1.26	1.86	17.92	0.022
K1: There are two sets of teeth during the lifetime	−1.24	2.67	15.02	0.059

**Table 5:** CFA for the attitude domain.

Figure	Items	Factor Loading	Reliability (Raykov’s Rho)
Daily Oral Hygiene	A1. Brushing teeth twice a day improves oral hygiene A6. Keeping your teeth clean and healthy is beneficial to your health	0.923 0.972	0.95
Oral Hygiene Habits	A4. Improper brushing leads to gum disease A8. Sweets retention leads to tooth decay A2. Brushing with fluoride toothpaste prevents tooth decay	0.967 0.987 0.956	0.98
Oral Hygiene Assumptions	A3. Dentists care only about treatment and not prevention A5. Gum bleeding denotes gum infection A7. Scaling is harmful for gums	0.981 0.944 0.893	0.97

**Table 6:** CFA for behaviour domain.

Factors	Items	Factor Loading	Reliability (Raykov’s Rho)
Behaviour towardsteeth	B3. I give importance to my teeth as much as any part of my body B4. I have sensitive teeth B6. I brush my teeth twice daily	0.984 0.889 0.887	0.94
Behaviour towardsteeth health	B5. I use my teeth to open the caps of bottled drinks B7. I experience tooth aches while chewing food	1.258 0.618	0.87
Behaviour towardsteeth conditions	B1. I have bleeding gums during brushing B2. I do routine dental check-ups	0.996 0.970	0.98

**Table 7:** Fit indices for confirmatory factor models.

Factors	No of Items		Goodness	Fit	Indices			
		X^2^ (df)	*p*-Value	CFI	TLI	RMSEA	SRMR	AIC
Attitude	8	39 (7)	0.002	0.991	0.986	0.07	0.016	4593.4
Behaviour	7	28 (11)	0.003	0.990	0.981	0.07	0.031	4086

## Data Availability

The datasets used in this study are obtainable from the corresponding author on request.

## Abbreviations

IRT	item response theory
CFA	confirmatory factor analysis
KAB	knowledge, attitude, and behaviour
RMSEA	root mean square error of approximation
CFI	comparative fit index
TLI	Tucker–Lewis Index
MLR	multiple linear regression
SRMR	standardized root mean square residual
AIC	Akaike information criterion
